# Viral diversity of *Rhipicephalus microplus* parasitizing cattle in southern Brazil

**DOI:** 10.1038/s41598-018-34630-1

**Published:** 2018-11-05

**Authors:** William Marciel de Souza, Marcílio Jorge Fumagalli, Adriano de Oliveira Torres Carrasco, Marilia Farignoli Romeiro, Sejal Modha, Meire Christina Seki, Janaína Menegazzo Gheller, Sirlei Daffre, Márcio Roberto Teixeira Nunes, Pablo Ramiro Murcia, Gustavo Olszanski Acrani, Luiz Tadeu Moraes Figueiredo

**Affiliations:** 1 Virology Research Center, School of Medicine of Ribeirão Preto of University of São Paulo, Ribeirão Preto, 14049-900 SP Brazil; 2 MRC-University of Glasgow Centre for Virus Research, Glasgow, G61 1QH Scotland United Kingdom; 3 Universidade do Centro Oeste do Paraná, Guarapuava, 85015-430 PR Brazil; 4 Laboratório de Bioquímica e Imunologia de Artrópode, Institute of Biomedical Sciences, University of São Paulo, São Paulo, 05508-900 SP Brazil; 5 Center for Technological Innovation, Instituto Evandro Chagas, Ananindeua, 67030-000 PA Brazil; 6 Universidade Federal da Fronteira Sul, Passo Fundo, 99010-200 RS Brazil

## Abstract

Ticks are ectoparasites spread worldwide and are well known as vectors of many viruses of great importance to human and animal health. However, the viral diversity in ticks is still poorly understood, particularly in South America. Here we characterized the viral diversity present in *Rhipicephalus microplus* parasitizing cattle in the southern region of Brazil using metagenomics. Our study revealed the presence of viruses that had not been previously described in the region, including lihan tick virus (*Phenuiviridae* family) and wuhan tick virus 2 (*Chuviridae* family), as well as expands the biogeography of jingmen tick virus (*Flaviviridae* family) in Brazil. Also, we described three novel tymoviruses (*Tymovirales* order), named guarapuava tymovirus-like 1 to 3. We described the genomic and phylogenetic characterization of these viruses. Our study sheds light on the viral diversity of *Rhipicephalus microplus* in South America, and also expands the biogeography of tick viruses that were previously described only in Asia.

## Introduction

Ticks are haematophagous ectoparasites that live by feeding on vertebrates, and they are known to be vectors of important human and animal pathogens^1^. Currently, approximately 900 species of ticks have been identified and taxonomically classified into three families: *Argasidae*, *Ixodidae*, and *Nuttalliellidae*^2,3^. Viruses transmitted to humans by bites of infected ticks are found mostly in the family *Ixodidae*, especially in the genera *Ixodes*, *Haemaphysalis*, *Hyalomma*, *Amblyomma*, *Dermacentor* and *Rhipicephalus*^1^.

To date, at least 38 viral species are known to be transmitted by ticks. They are distributed in eight viral families: *Asfarviridae*, *Arenaviridae*, *Reoviridae*, *Rhabdoviridae*, *Orthomyxoviridae*, *Flaviviridae*, *Nairoviridae*, and *Phenuiviridae*^4,5^. Many tick-borne viruses are emerging and re-emerging pathogens, which have caused outbreaks of devastating and often fatal diseases, representing a serious public and animal health problem in many regions of the world. Thrombocytopenia syndrome virus, Heartland and Louping ill^4,6,7^ are examples of emerging viruses, while Crimean-Congo hemorrhagic fever, African swine fever virus and Nairobi sheep disease virus are re-emerging viruses^4^.

Recently, studies based on high-throughput sequencing (HTS) have revealed an unprecedented diversity of viruses in ticks, including new viral species classified into families *Rhabdoviridae*, *Reoviridae*, *Flaviviridae*, and orders *Bunyavirales* and *Mononegavirales*^8–10^. Also, novel viruses that have not been classified can represent important pathogens in human or veterinary health, as well as viruses that may be regarded as commensals^8,11–14^.

Currently, twenty-eight species of *Ixodidae* ticks have been shown to be distributed in South America, including 21 *Amblyomma*, two *Dermacentor*, two *Haemaphysalis*, one *Boophilus*, one *Ixodes* and one *Rhipicephalus* species^3^. Despite the widespread of ticks in this region and the high importance of tick-borne viruses, there are only two viruses associated with ticks described in South America: Cacipacoré virus and Mogiana tick virus (MGTV)^15–17^.

Here, we applied a metagenomics approach to determine the diversity of viruses present in *Rhipicephalus microplus* parasitizing cattle in the southern region of Brazil. In addition to the virome analysis, we performed experimental infections using different host cell lines to assess the *in vitro* host range of the viruses that were identified.

## Results

The HTS analysis of six pools of ticks (~50 per pool) and five pools of bovine blood (4 to 15 per pool) generated a total of 7,634,294 to 34,753,840 paired-end reads with 68.56% to 95.47% of bases ≥ Q30 with a base call accuracy of 99.90% (Supplementary Table 1). The first step of the MetaViC pipeline removed between 45% to 81% paired-end reads categorized as non-viral sequences. After *de novo* assembly, the viral contigs represented 0.30 to 24% of all contigs in each library (Supplementary Table 2). A total of 76 to 99% of reads were classified as eukaryote and bacteria, and unclassified reads were identified in 0.2 to 4% total reads in four sample pools (Supplementary Table 2). In a subsequent analysis of the viral contigs, we identified three previously known viral genomes: jingmen tick virus (JMTV), lihan tick virus (LITV) and wuhan tick virus 2 (WTV-2), as well as three previously uncharacterized viruses related with *Tymovirales* order. The analyses of each virus species are described below.

### Jingmen tick virus (*Flaviviridae* family)

**Figure 1:**
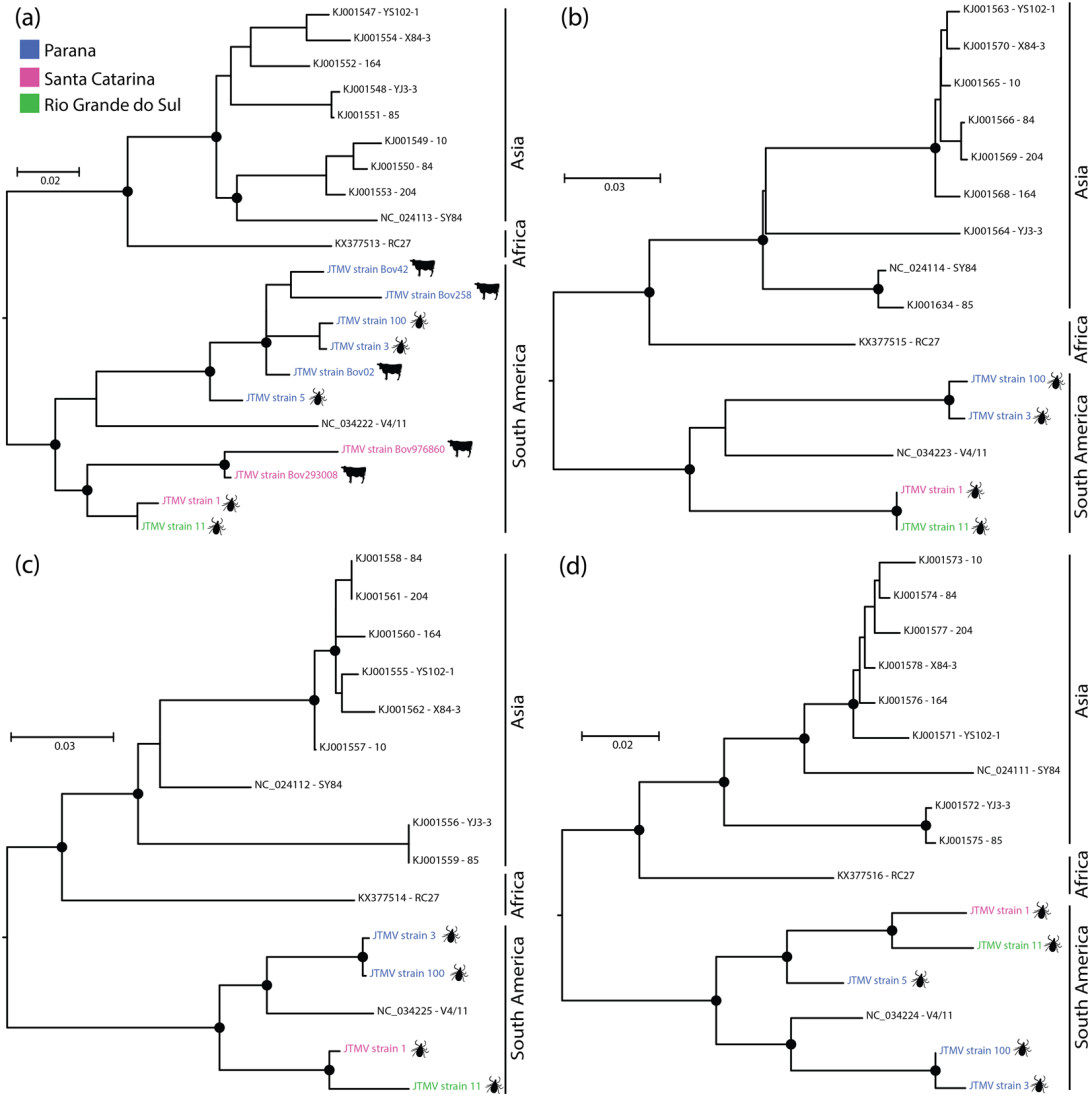
Maximum likelihood phylogenies of new strains of Jingmen tick virus. Phylogenies were constructed on nucleotides alignments, (**a**) segment 1 (NS5-like) based on TN + F + I nucleotides substitution model, (**b**) segment 3 (NS3-like) based on TIM3 + F + I nucleotides substitution model, (**c**) segment 2 (Glycoprotein) based on TIM3 + F + G4 nucleotides substitution model and (**d**) segment 4 (VP2-VP3) based on TNe + I nucleotides substitution model. Phylogenies are midpoint rooted for clarity of presentation. The scale bar indicates evolutionary distance in numbers of substitutions per nucleotides sites. Taxons are colored according to geographical location, and the hosts were denoted with a silhouette. The legends for the colors are shown on the left. The black circles indicate the main nodes with maximum likelihood bootstrap support levels above 75% bootstrap replicates.

We identified three nearly complete sequences and five partial genomes of JMTV, a unique segmented flavivirus-like species (Supplementary Figs 1 and 2, Fig. 1). This virus was identified in RNA derived from cattle-infesting ticks and cattle blood. We found four positive-sense single-stranded RNA segments of JMTV, named as segments 1 to 4, with 2,994; 2,771; 2,715 and 2,672 nucleotides (nt) in length, respectively (Supplementary Fig. 2a). These four segments of JTMV encode five proteins^9^. Based on BLAST analysis, we identified that this virus was 97 to 98% identical at the amino acid level with JMTV and MGTV^9,15,16^. The mean coverage of each segment was 6 to 12 × (segment 1), 6 to 16 × (segment 2), 5 to 31 × (segment 3) and 10 to 71 × (segment 4). To determine the frequency and geographic distribution of JMTV in cattle, the individual cattle samples were screened by RT-PCR as previously described^9^. JMTV was detected in 67% (4/6) of tick pools and 14% (5/36) of bovine sera. Interestingly, our phylogenetic analysis revealed that JMTV strains identified in cattle were closely related to strains from ticks sampled from the same geographical regions (Fig. 1a–d). Also, phylogenetic trees showed that the JMTV circulating in Brazil is a possible different genotype than those previously described in Africa and Asia (Fig. 1a–d).

We attempted to isolate JMTV strain Bov976860 in invertebrate and vertebrate cell lines. The virus was serially passaged three times, and the segment 1 genome (NS5-like) of JMTV was detected by RT-PCR at day six post-infection in the first and second passage on C6/36 cells, and in the first passage on BME26 cells. A gradual detachment of the cell layer was observed in C6/36 and BME26 cells during the first passage, although a typical cytopathic effect (CPE) was not observed. Collectively, these results showed that JMTV is present in *Rhipicephalus microplus* ticks and cattle infested by them in the southern region of Brazil.

### Lihan tick virus (*Phenuiviridae* family)

**Figure 2:**
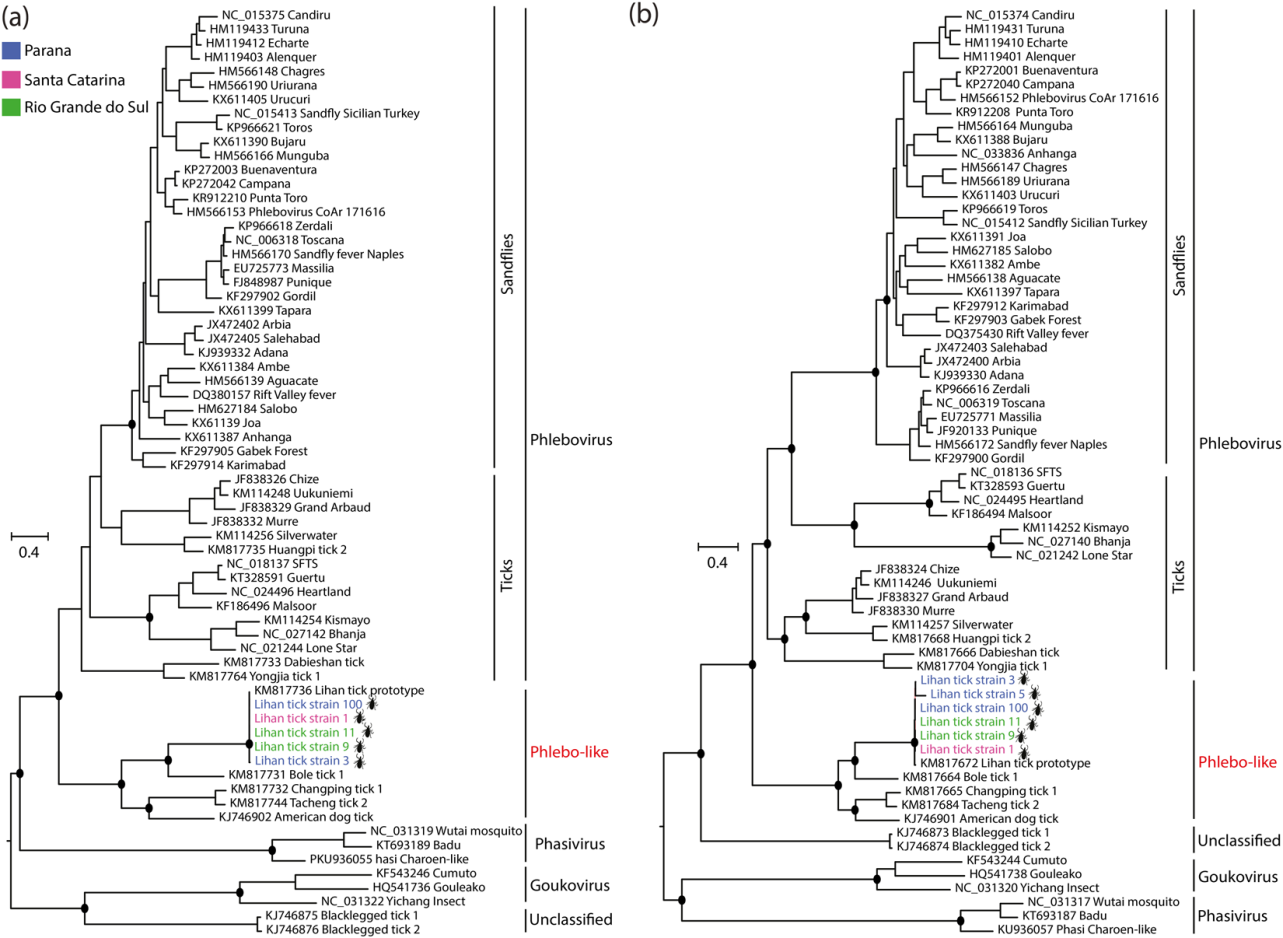
Maximum likelihood phylogenies of new strains of Lihan tick virus into *Phenuiviridae* family. Phylogenies were constructed on amino acids alignments sequences of nucleoprotein (**a**) and RNA-dependent RNA polymerase (**b**) based on LG + I + G4 amino acids substitution model. Phylogenies are midpoint rooted for clarity of presentation. The scale bar indicates evolutionary distance in numbers of substitutions per amino acids sites. Taxons are colored according to geographical location, and the host was denoted with a silhouette. The legends for the colors are shown on the left. The black circles indicate the main nodes with maximum likelihood bootstrap support levels above 75% bootstrap replicates.

We identified a nearly complete sequence of the small (S) and large (L) segments of two strains and partial genomes of four strains of a unique species of a phlebovirus-like virus in the tick pools. The nearly full L and S segments presented 6,495 and 1,535 nucleotides in length, respectively (Supplementary Fig. 2b), encoding the RNA-dependent RNA polymerase (RdRP) and the nucleoprotein (N), respectively. The mean coverage of each segment was 6–13 × to S segment and 6–22 × to L segment. Intriguingly, we did not detect the homologous medium segment (M), which encodes for the glycoprotein precursor (GPC) and is present in the majority of phleboviruses (*Phenuiviridae* family). Based on BLAST analysis, we identified that this virus shared 97 to 98% amino acid identities with a LITV described in *Rhipicephalus microplus* collected in China^8^. Using RT-PCR, we confirmed the HTS results, which LITV was present in 5 out of 6 of tick pools from all states sampled in the southern region of Brazil. However, not a single sample derived from cattle was positive for this virus. Phylogenetic analysis of both RdRP and N proteins reveals that LITV strains form a monophyletic group with other phlebovirus-like viruses with uncertain M segment, showing a clade that is basal to the *Phlebovirus* genus in the *Phenuiviridae* family (Fig. 2a,b). We tried to isolate LITV strain 9 in invertebrate and vertebrate cell lines. The virus was serially passaged three times, and the L segment genome (RdRP) of LITV was detected by RT-PCR at six days after inoculation only in the first and second passage on C6/36. However, LITV was undetected in BME26 cells and vertebrates cell lines. Collectively, these results showed that LITV is circulating in *Rhipicephalus microplus* ticks throughout the southern region of Brazil.

### Wuhan tick virus 2 (*Chuviridae* family)

We identified two nearly complete sequences, and four partial genomes of WTV-2, a virus that belongs to a recently described family referred to as *Chuviridae*. These viruses shared 97 to 98% amino acid identity with WTV-2 identified in *Rhipicephalus microplus* collected in China^8^. The genome organization of WTV-2 described here are linear single-stranded negative RNA with ~10 kb in length, which encodes a glycoprotein, nucleoprotein, and polymerase protein (Supplementary Fig. 2c). The mean coverage of the genomes was between 14 to 44×. The RT-PCR confirmed the HTS results, which showed that 5 out 6 pools were positive to WTV-2 in tick pools from all states sampled in the southern region of Brazil.

**Figure 3:**
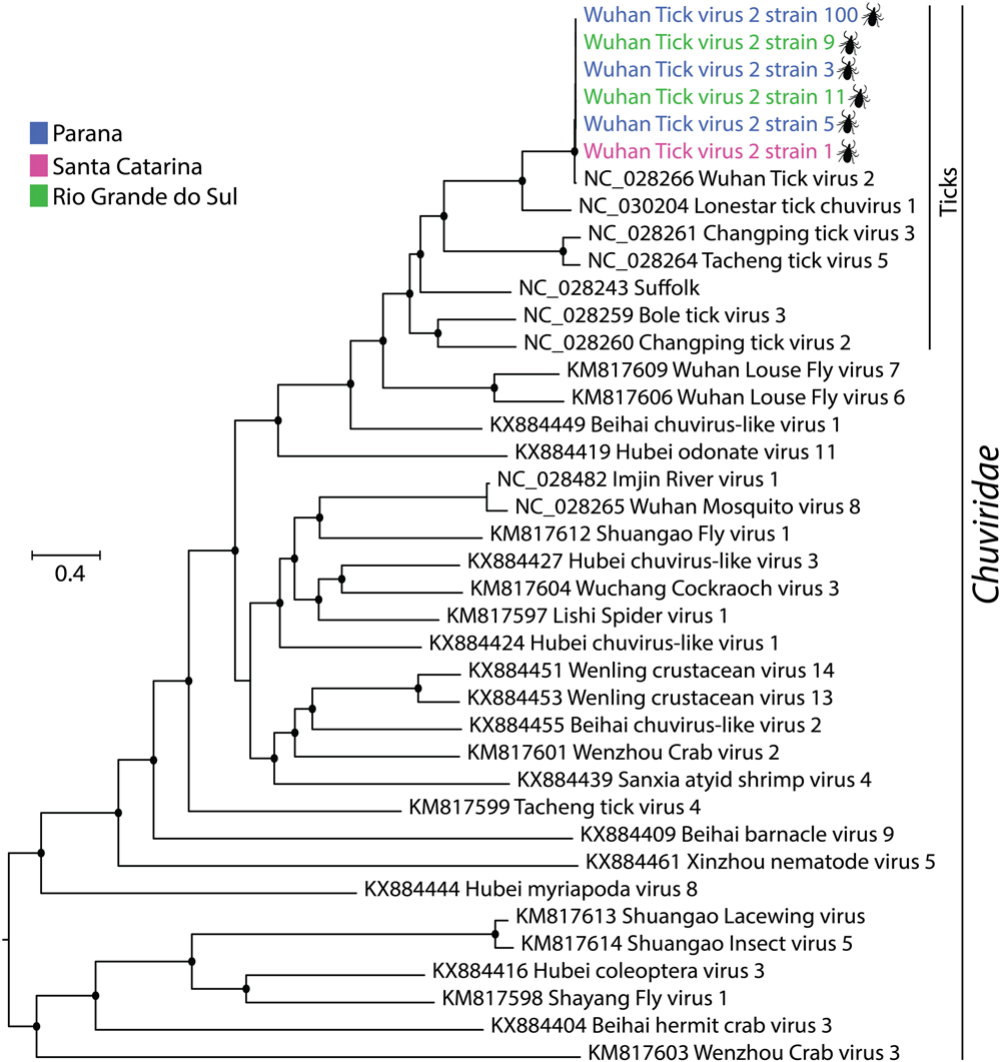
Maximum likelihood phylogeny of new strains of Wuhan tick virus 2 into *Chuviridae* family. Phylogeny was constructed on amino acids alignments sequences with RNA-dependent RNA polymerase based on LG + I + G4 amino acids substitution model. Phylogeny was midpoint rooted for clarity of presentation. The scale bar indicates evolutionary distance in numbers of substitutions per amino acids sites. Taxons are colored according to geographical location, and the host was denoted with a silhouette. The legends for the colors are shown on the left. The black circles indicate the main nodes with maximum likelihood bootstrap support levels above 75% bootstrap replicates.

However, we did not find any positive serum sample from cattle. Interestingly, phylogenetic analysis revealed that all the WTV-2 strains form a monophyletic clade within the *Chuviridae* family, grouping with other chuviruses that have also been described in ticks (Fig. 3). As above, we attempted to isolate and grow the WTV-2 strain 11 in invertebrate and vertebrate cell lines. The virus was serially passaged three times, and the partial sequence of the polymerase ORF of the virus was detected by RT-PCR at six days after inoculation in the first and second passage on C6/36 and on the first passage on BME26, Vero, Vero E6, HEK-293, LLC-PK1, and UMNSAH/DF. The ability of the virus to grow in cell lines was limited to first passages on vertebrate and invertebrate cells without gradual detachment of any of the cell line layers. It is also important to consider that the viral RNA detected by PCR can be a result of residual viral RNA from the inoculum. Collectively, these results showed that WTV-2 is widespread in *Rhipicephalus microplus* ticks in the southern region of Brazil.

### Novel tymoviruses (*Tymovirales* order)

**Figure 4:**
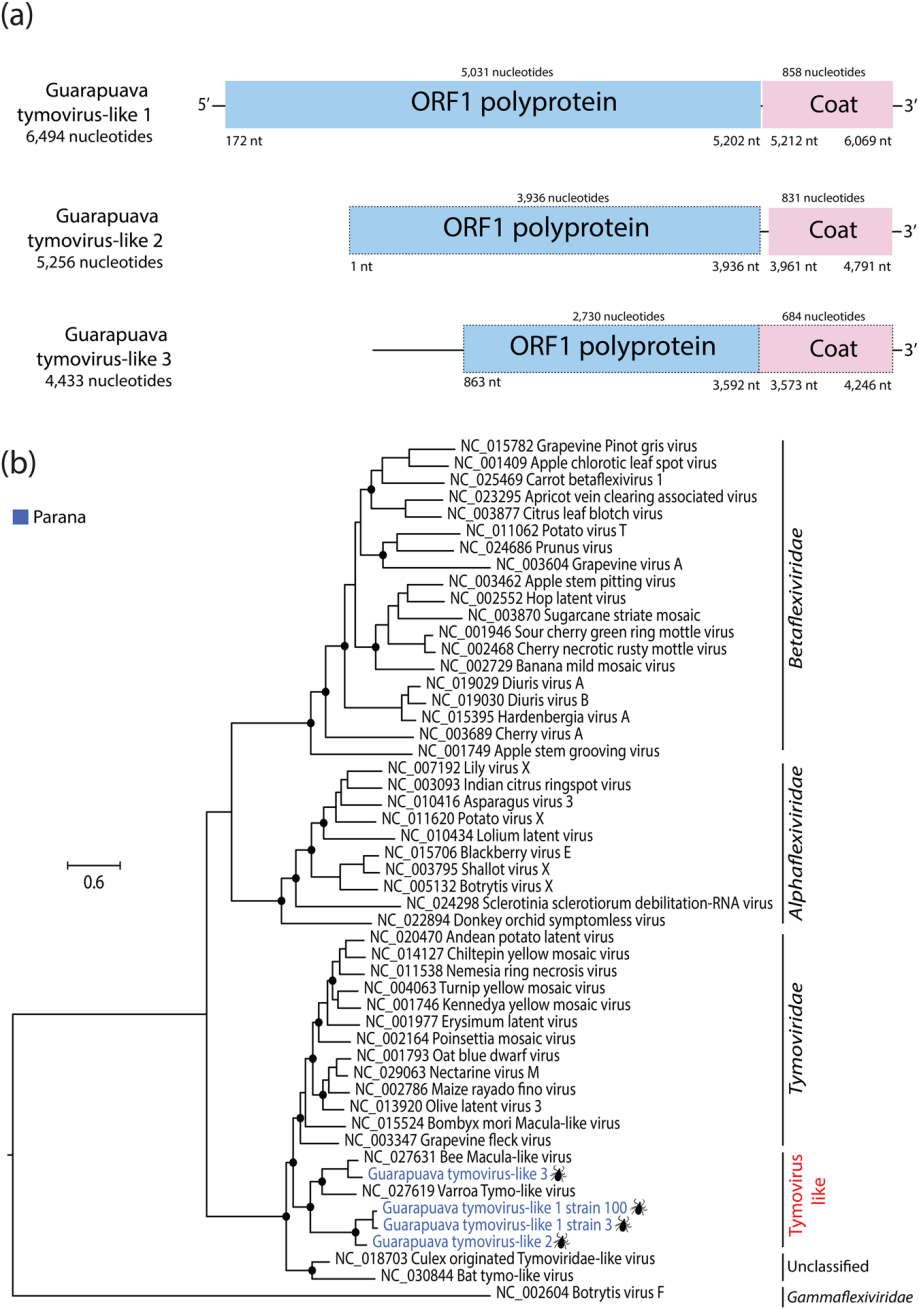
Genome organization of novel tymoviruses (**a**). The ORF1 polyprotein and coat gene are shown in blue and pink, respectively. The respective nucleotides positions of elements in genome are shown at the bottom of figures. Dashed-lines indicate the incomplete gene. (**b**) Maximum likelihood phylogeny of novel tymoviruses into *Tymovirales* order. Phylogeny was constructed on amino acids alignments sequences with ORF1 polyprotein based on LG + I + G4 amino acids substitution model. Phylogeny was midpoint rooted for clarity of presentation. The scale bar indicates evolutionary distance in numbers of substitutions per amino acids sites. Taxons are colored according to geographical location, and the host was denoted with a silhouette. The legends for the colors are shown on the left. The black circles indicate the main nodes with maximum likelihood bootstrap support levels above 75% bootstrap replicates.

We identified two nearly complete sequences and two partial genomes of three novel species of tymoviruses-like in pools of *Rhipicephalus microplus* collected in Paraná State, which were tentatively named as guarapuava tymovirus-like 1 (GTV-like 1), guarapuava tymovirus-like 2 (GTV-like 2) and guarapuava tymovirus-like 1 (GTV-like 3). The nearly complete genome of GTV-like 1 was 6,625 nt long, and the partial genome of GTV-like 2 and GTV-like 3 were 5,256 nt and 4,433 nt long, respectively (Fig. 4a). The mean coverage of viruses were 36,255× (GTV-like 1), 21,844× (GTV-like 2), and 6,713× (GTV-like 3). The genome of these viruses exhibits the typical genome organization associated with members of the *Tymoviridae* family, with two ORFs that encode for a polyprotein and capsid (Fig. 4a). Phylogenetic analysis of the ORF1 polyprotein sequence showed that the two strains of GTV-like 1, the GTV-like 2 and the GTV-like 3 form a monophyletic group with other tymovirus-like identified in arthropods, which is basal to the *Tymoviridae* family (Fig. 4b). Furthermore, GTV-like 1 and GTV-like 2 shared only 31% to 43% amino acid identities with Bee Macula-like virus and Varroa Tymo-like virus, and GTV-like 3 shared 79% and 81% amino acid identities with Bee Macula-like virus in the non-structural polyprotein and capsid sequences. Based on our analysis and the species demarcation criteria of the International Committee on Taxonomy of Viruses (ICTV) to the *Tymovirales* order, we propose that the GTV-like 1, 2 and 3 might constitute a new family into *Tymovirales* order. Based on HTS approach, we detected GTV-like 1, 2 and 3 in ticks from the south of Brazil in two pools (WM100 and WM3), and we did not find any positive in cattle sera. Collectively, these results showed that novel lineage of tymoviruses, comprised of viruses referred to as GTV-like 1, 2 and 3, is circulating in the central region of Paraná State in *Rhipicephalus microplus* ticks.

## Discussion

Viruses are the most abundant infectious agents present in all domains of life, and invertebrates comprise the vast majority of the *Animalia* kingdom^13,18^. Recently, metagenomic studies have shown that invertebrates exhibit a greater viral diversity than vertebrates, and such ‘virosphere’ might in part reflect the enormous species number and diversity of arthropods^8,9,12,13,18,19^. Ticks are globally spread ectoparasites that act as vectors of human and animal pathogens because they feed on a wide range of animal hosts, including humans, in both wild and urban areas^4^. Here, we explored and described the virome of *Rhipicephalus microplus* ticks from cattle in Brazil, including the characterization and molecular epidemiology of viruses from *Flaviviridae*, *Phenuiviridae*, and *Chuviridae* as well as the *Tymovirales* order.

JMTV is a recently discovered virus identified in *Rhipicephalus microplus* ticks in China^9^. This virus possesses a genome organization composed of four segments, two of which are related to the nonstructural genes of the *Flavivirus* genus, suggesting the first evidence of a segmented flavivirus-like species. Based on recent studies where other viruses associated with JMTV were identified, the genus *Jingmenvirus* was proposed within the *Flaviviridae* family^20,21^. Our genomic analysis revealed that JMTV and MGTV, another virus previously detected in *Rhipicephalus microplus* ticks in Central-West and Southeast region of Brazil, shared 97% to 99% nucleotide identity in all segments, suggesting that they might be the same viral species^9,15,16^. The frequency of JTMV in ticks collected in Brazil was similar to that previously described in China, but we observed a higher prevalence of JMTV in bovine samples in our study compared to the previous report in China with ~14% *versus* 3.44%, respectively^9^. Therefore, considering MGTV as the same viral species as JMTV, our results expanded the geographic distribution of JMTV. We also describe for the first time the presence of JMTV in cattle in the American continent. This finding highlights the need for further studies to elucidate the host range of JMTV. Interestingly, the phylogenetic trees showed that the JMTV strains described in Brazil clustered within a monophyletic clade that is distinct from the strains identified in China and Africa, suggesting that JTMV strains circulating in Brazil probably belong to a new genotype^9,21^.

We attempted to isolate JMTV, but it was unsuccessful due to the lack of plaque formation. Furthermore, we could not visualize CPE on different cell lines. However, this is not surprising because issues to grow JMTV in cell lines have been previously reported^9,15^. On the other hand, although we performed experimental infections on several invertebrate and vertebrate cell lines, JMTV could only be detected in the invertebrate cells C6/36 and BME26. However, further studies must be conducted to elucidate the role of JMTV as a potential tick-borne pathogen.

LITV is a segmented negative-stranded RNA virus with a linear genome classified into the *Phenuiviridae* family. The strains of LITV identified in our study shared high levels of identity at the amino acid level with the prototype virus described in *Rhipicephalus microplus* in China^8^. Although we recovered a nearly complete genome of both large and small segments of LITV, we were unable to identify any sequence with similarity to segments that encode the GPC encoded in the medium segment. The same observation was previously described for the prototype virus^8^. Our molecular epidemiology analysis showed that LITV has a high-frequency distribution in ticks in the southern region of Brazil, while it was unable to be detected in cattle. From the viral propagation attempts, we have detected the genome of LITV only in invertebrate cell lines, suggesting that replication of LITV might be restricted only to invertebrate host, but the attempts of viral isolation were unsuccessful in this study. Interestingly, our phylogenetic analysis showed that LITV strains were clustered in a monophyletic group with other phlebovirus-like viruses with an unrecognized medium segment, which can be a potential new genus in *Phenuiviridae* family. Recent studies have described some new phlebovirus-like viruses that, like LITV, apparently do not harbor a medium segment. These viruses were detected in ticks collected in the United States of America (USA) and in China^8,11,22^. Several explanations have been suggested in order to understand the detection of a phlebovirus-like virus with only the small and large segments, including: (i) these viruses may have an atypical M segment that is undetected by HTS approach due to its great sequence divergence; (ii) they could actually encode only for the large (RdRP) and small (N protein) proteins while coping with other means for cellular entry, since they do not code for an envelope glycoprotein; (iii) small and large segments may exist in an episome-like form in tick cells, thus may not form an infectious virion, but may instead use transovarial transmission or some other means as a vehicle for their dissemination to new hosts^11^. Also, LITV could be a retrotransposon-like or an endogenous viral element, which is unlikely because we showed that LITV genomes could be detected in invertebrate cell lines after serial passages, suggesting that the virus is replicating *in vitro*. We also did not detect the LITV viral genome in BM26, a tick lineage. Unfortunately, we were unable to isolate LITV by plaque-forming assay due to the inability of the virus to replicate in the vertebrate cell lines we used. Therefore, further studies should elucidate an atypical M segment or a new alternative mechanism for viruses with different glycoprotein coding strategies in the *Bunyavirales* order.

The *Chuviridae* is a putative novel viral family with representatives of both segmented and unsegmented viruses^8^. Here, we expanded the geographical distribution of this family, describing a wide circulation of WTV-2 in *Rhipicephalus microplus* ticks in the southern region of Brazil. This virus is very similar at the amino acid level with the prototype species and formed a monophyletic clade with other chuviruses described in ticks from China and USA^8,13^. This viral family is constituted only by viruses described in invertebrates, but we detected the genome of WTV-2 in invertebrate and vertebrate cell lines, as observed in our viral propagation attempts, but the attempts of viral isolation were unsuccessful in this study, and the genome detection can be residual viral RNA from inoculum.

Our virome analysis also identified three novel viruses characterized as having a similar genome organization from viruses of the *Tymoviridae* family (*Tymovirales* order), a predominantly plant-infecting virus family and transmitted by phloem-sucking insects^23^. These viruses, tentatively named GTV-like 1, 2 and 3 constitute a divergent monophyletic group with Bee Macula-like virus and Varroa Tymo-like virus, both unassigned tymoviruses^24^. The Bee Macula-like virus and Varroa Tymo-like have been observed with high prevalence in honeybees (*Apis mellifera L*.) and varroa mites (*Varroa destructor*) in Europe and USA, but the probable vector is *Varroa destructor*^24^. Although these viruses could be considered as contaminating plant viruses in our tick samples, the *Varroa destructor* is an obligate parasite that only feeds on bee hemolymph and has no contact with plants, suggesting that GTV-like 1, 2 and 3 could also be invertebrate viruses that share the same common ancestor. However, further studies are needed to reveal the role of these viruses in the pathogenesis of invertebrate hosts.

It has already been reported that the known viral diversity is under-sampled and underestimated^13,14,18^. Here, although the sample size of ticks used in this study was relatively small, we extended the current viral diversity with the notable characterization of viruses from *Rhipicephalus microplus* and its parasitized cattle in the southern region of Brazil. We supposed that these viruses identified in our study and previously reported in Asia could result from a deep association or co-evolution with *Rhipicephalus microplus*, and possibly can be found worldwide in subtropical and tropical regions in this tick species^25,26^. This region comprises three different states, covers 576,774 km², and is considered a major production hub of bovine meat for export. Finally, our results showed that viruses from ticks could be spread on multiple continents and also highlight the role of ticks as important viral reservoirs in South America.

## Methods

### Samples

**Figure 5:**
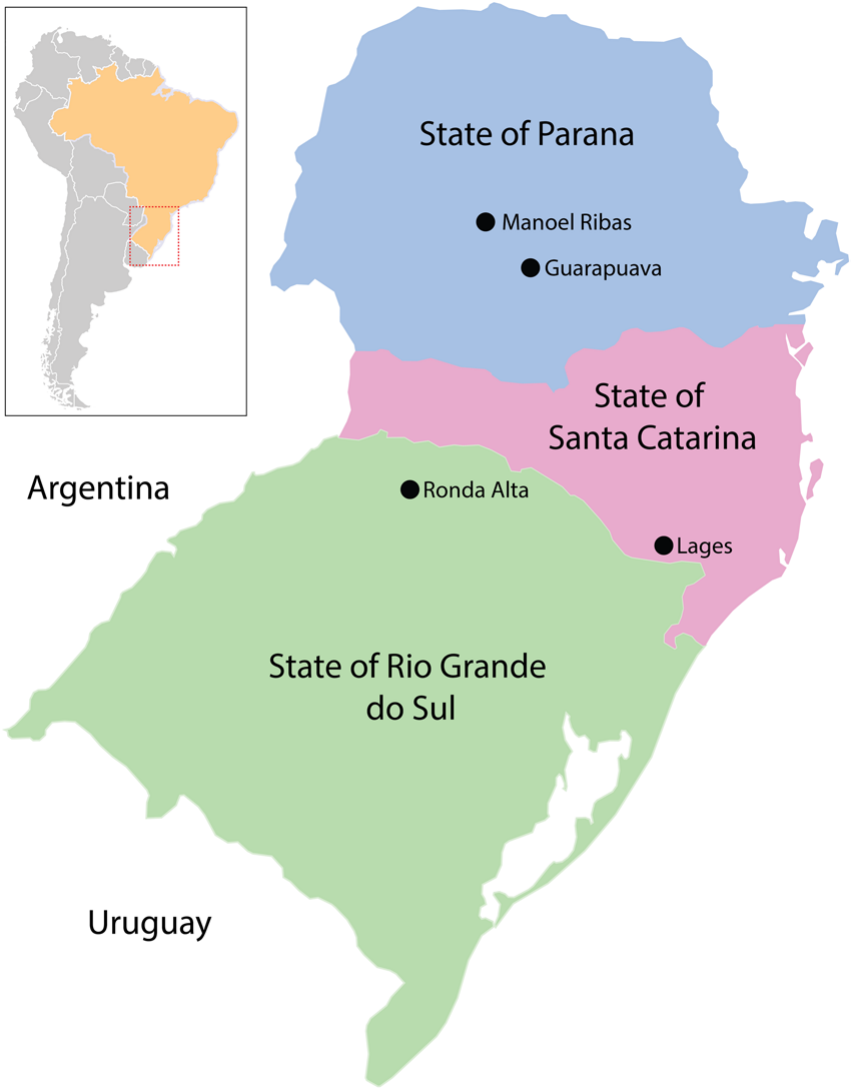
Map of sample collection sites in the southern region of Brazil. Black dots represent the name of the collection sites in each state.

Paired samples (engorged ticks and blood) from 36 cattle were collected between October 2015 to June 2016 from four locations in the southern region of Brazil. The sites of collection were cattle farms in the municipalities of Guarapuava and Manoel Ribas in Paraná State, Lages city in Santa Catarina State, and Ronda Alta county in the Rio Grande do Sul State, all from the southern region of Brazil (Fig. 5 and Supplementary Table 1). After collection, all ticks were transported to the laboratory at the Universidade Estadual do Centro-Oeste do Paraná (UNICENTRO). Ticks were identified based on morphological features^27^. All samples were stored at −80 °C. All procedures, protocols, and methods performed in this study were approved based on guidelines and regulations of animal research by the Ethics Committee for Animal Research of the UNICENTRO (001/2016).

### Sample preparation

Samples were placed in pools of same species (tick or cattle), date and collection site. Tick pools (~50 engorged ticks per pool) were homogenized with a mortar and pestle in 1 ml of Hank’s buffered salt solution (HBSS). Ticks and bovine sera pools were centrifuged at 10,000 g for 10 minutes at 4 °C, and supernatants were filtered using 0.22 μm-pore-size polyvinylidene difluoride filters (TPP, Switzerland) to minimize the presence of cells, debris, and bacteria. Subsequently, the pools were ultracentrifuged at 180,000xg for 3 hours at 4 °C in a 30% sucrose solution (Thermo Fisher Scientific, USA), and resuspended in 500 μl of HBSS. To remove the naked DNA and RNA, 200 μl of the resuspended pellet from each pooled sample were digested in a cocktail with 20U of Turbo DNase (Life Technologies, USA), 25U of benzonase (Sigma-Aldrich, USA), and 0.1 mg/ml of RNase A (Life Technologies, USA) at 37 °C for 2 hours in 20 μl of 10X DNase buffer (Life Technologies, USA). Then, the capsid-protected viral genomes were extracted with a QIAamp viral RNA mini kit (Qiagen, USA). Subsequently, RNA was quantified using a Qubit® 2.0 Fluorometer (Invitrogen, USA) and the purity and integrity of RNA samples were measured using an Agilent 2100 Bioanalyzer (Agilent Technologies, USA).

### Genome sequencing and viral assembly

cDNA was generated from total RNA using the Superscript II kit (Invitrogen, USA) according to the manufacturer’s instructions. cDNAs were prepared for high-throughput sequencing using the TruSeq RNA Universal (Illumina, USA) protocols and standard multiplex adaptors. A paired-end, 150-base-read protocol in the RAPID module was used for sequencing on an Illumina HiSeq. 2500 instrument as recommended by the manufacturer. Sequencing was performed at the Life Sciences Core Facility from University of Campinas, Brazil. The resulting sequencing reads were assembled *de novo* using the metaViC pipeline (https://github.com/sejmodha/MetaViC)^28^. This pipeline is divided into two major components. The first step of the cleaning pipeline is to remove the adapters and short reads (<80 nucleotides) using Trim Galore (https://www.bioinformatics.babraham.ac.uk/projects/trim_galore/). The ribosomal RNA (rRNA) sequences are removed using RiboPicker^29^. After this, DIAMOND^30^ can be run for each read against the refseq protein database for each file. Krona charts^31^ are generated for each DIAMOND output file that describes the read based classification of the sample. The DIAMOND results are then converted to a BLAST tabular output and the Genbank Identifier (GI) column from the output is extracted. GIs are mapped back to NCBI taxonomy databases to extract the corresponding taxonomy and division ID. In our case, we are only interested in the sequences that match viruses, environmental sequences and the sequences that do not match anything in the database. Therefore, any read that is matching protein sequences originating from bacteria, invertebrates, mammals, rodents, phages, plants, vertebrates, primates, and synthetic constructs are identified based on the division ID and are filtered from the sample files. Finally, the sample files are properly paired using Prinseq-lite.pl^32^. These reads can then be submitted to the next stage of the pipeline. Sequence reads from the previous step of the pipeline can be submitted to this script that carries out the *de novo* assembly using SPAdes^33,34^ and IDBA-UD^35^ with the k-mer values 31, 55, 77, and 99 for SPAdes and a range of the k-mer values starting with minimum k of 31 to maximum k of 99 for IDBA-UD.

The assembled contigs from two assembly tools are then merged using GARM^36^, an assembly merging pipeline that uses mummer to find overlaps between two assemblies and joins them. Reads are aligned back to the contigs (>200 nucleotides), and supercontigs using bowtie2^37^ and unmapped reads are extracted using bam2fastq. In order to check the assembly quality, a QUAST^38^ analysis is performed for each contig assembly and supercontigs. Contigs longer than a length of 200 and supercontigs generated by GARM are merged and classified using DIAMOND against refseq protein database. Krona tools are used to create interactive HTML output to visualize the BLAST tabular output formatted results generated by DIAMOND. After, we also performed the reference genome with viruses found using Bowtie2^37^.

### Viral genomic characterization

Viral genomes were evaluated regarding size and ORF prediction with Geneious 9.1.2 (Biomatters, New Zealand). Protein sequences were submitted to the TOPCONS web server^39^ for identification of transmembrane regions and signal peptide, and to NetNglyc 1.0 Server (http://www.cbs.dtu.dk/services/NetNGlyc/) for identification of glycosylation sites. Annotations of protein domains were performed with InterPro 60.0^40^.

### Phylogenetic analysis

Maximum likelihood (ML) phylogenetic trees were inferred using amino acids and nucleotides alignments of viral genomes described in this study with representative members of each viral family. Multiple sequence alignments (MSA) were generated using RevTrans 2.0^41^, and manually edited to removing all ambiguously aligned regions. ML trees were inferred using IQ-TREE version 1.4.3 applying 1,000 bootstraps, substitution models were determined by the best-fit model based on Bayesian Information Criterion considered 144 reversible amino acids substitution models and 88 nucleotides substitution models^42,43^. Phylogenetic trees were visualized using FigTree v.1.4.2.

### Frequency of viruses

To determine the authenticity and frequency of viral sequences in tick pools and individual cattle samples, we designed primer sets to specifically amplify for each virus identified by HTS (Supplementary Table 3). Then, the viral RNA of individual cattle samples and tick pools were extracted using the QIAamp viral RNA extraction kit (Qiagen, Germany) and submitted to RT-PCR to LITV and WTV-2 using SuperScript™ III One-Step RT-PCR System with Platinum™ Taq DNA Polymerase (Thermo Fisher Scientific, USA), following the manufacturer’s instructions. Cycling conditions were: 50 °C for 30 minutes, 94 °C for 2 minutes followed by 30 cycles at 94 °C for 30 seconds, 50 °C for 30 seconds and 68 °C for 1 minute, followed by a final extension of 72 °C for 5 minutes. The RT-PCR to JMTV was performed as previously described^9^. Amplicons were visualized by gel electrophoresis in 1.5% agarose gels. All PCR products were verified by dideoxy sequencing using ABI 3730 genetic analyzer (Applied Biosystems, USA).

### Cells

Various vertebrate and arthropod cell lines were used in experimental infections including: C6/36 (*Aedes albopictus*), BME26 (*Rhipicephalus microplus*), BHK-21 (baby hamster kidney), LLC-PK1 (pig kidney cell line), MDBK (bovine kidney), UMNSAH/DF (chicken embryo fibroblast), HEK-293 (human embryonic kidney), Vero (African green monkey kidney) and Vero E6 (a Vero cell line deficient for type I interferon). C6/36 and BME26 cells were propagated as previously described using Leibovitz’s L-15 medium supplemented using 10% heat-inactivated fetal bovine serum, 50 mg/ml of gentamicin and 2 mg/ml of amphotericin B, and maintained at 28 °C or 34 °C, respectively^44,45^. Vertebrate cell lines (BHK-21, MDBK, Vero, Vero E6 and HEK-293 and UMNSAH/DF) were propagated as previously described using D-MEM containing 10% fetal bovine serum (FBS), 100 U/ml of penicillin and 100 μg/ml streptomycin at 37 °C with 5% CO_2_^46–49^.

### Experimental infections

Virus samples were filtered through a 0.22-μm filter, and 250 μl was inoculated onto cell monolayers in T25 flasks. Flasks were gently rocked for 1 hour at 37 °C before 7 ml of the respective culture media containing 4% FBS was added. Inoculated cells were incubated for six days. Viruses were passaged three times in each cell line, and for each passage, RNA was extracted from cells and supernatant. Virus infection was assessed by RT-PCR and Sanger sequencing, as described above.

## Electronic supplementary material

Supplementary Information

## Data Availability

All sequence reads generated in this project are available under the NCBI Short Read Archive (SRA) under accessions SRR6848868-SRR6848875 (BioProject ID: PRJNA438342) and all consensus virus genome sequences have been deposited in GenBank (accession numbers: MH155881-MH155927).
