# Supplementary material for: Viral diversity of Rhipicephalus microplus parasitizing cattle in southern Brazil
Source: Sci Rep. 2018 Nov 5;8:16315. doi: 10.1038/s41598-018-34630-1 (PMC6218518; doi:10.1038/s41598-018-34630-1)
Supplement: Supplementary file 1 — Supplementary Information [file 41598_2018_34630_MOESM1_ESM.pdf]

Viral diversity of *Rhipicephalus microplus* parasitizing cattle in southern Brazil

William Marciel de Souza[1,2,*], Marcílio Jorge Fumagalli[1], Adriano de Oliveira Torres Carrasco[3], Marilia Farignoli Romeiro[1], Sejal Modha[2], Meire Christina Seki[3], Janaína Menegazzo Gheller[3], Sirlei Daffre[4], Márcio Roberto Teixeira Nunes[5], Pablo Ramiro Murcia[2], Gustavo Olszanski Acrani[6,+] and Luiz Tadeu Moraes Figueiredo[1,+].

[1]Virology Research Center, School of Medicine of Ribeirão Preto of University of São Paulo, Ribeirão Preto, 14049-900, SP, Brazil.

[2]MRC-University of Glasgow Centre for Virus Research, Glasgow, G61 1QH, Scotland, United Kingdom.

[3]Universidade do Centro Oeste do Paraná, Guarapuava, 85015-430, PR, Brazil.

[4]Laboratório de Bioquímica e Imunologia de Artrópode, Institute of Biomedical Sciences, University of São Paulo, São Paulo, 05508-900, SP, Brazil.

[5]Center for Technological Innovation, Instituto Evandro Chagas, Ananindeua, 67030-000, PA, Brazil.

[6]Universidade Federal da Fronteira Sul, Passo Fundo, 99010-200, RS, Brazil.

[+]These authors contributed equally to this work.

* Corresponding author: wmarciel@usp.br or wmarciel@hotmail.com

Supplementary Figures

Supplementary Figure 1. Maximum likelihood phylogeny showing the new strains of Jingmen tick virus into *Flaviviridae* family. Phylogenies were constructed on amino acids alignments sequences of NS3-like (left) and NS5-like (right) based on LG+I+G4 amino acids substitution model. Phylogenies are midpoint rooted for clarity of presentation. The scale bar indicates evolutionary distance in numbers of substitutions per nucleotides sites. Taxons are colored according to geographical location and the host was denoted with a silhouette. The legends for the colors are shown on the left. The black circles indicate the main nodes with maximum likelihood bootstrap support levels above 75% bootstrap replicates.

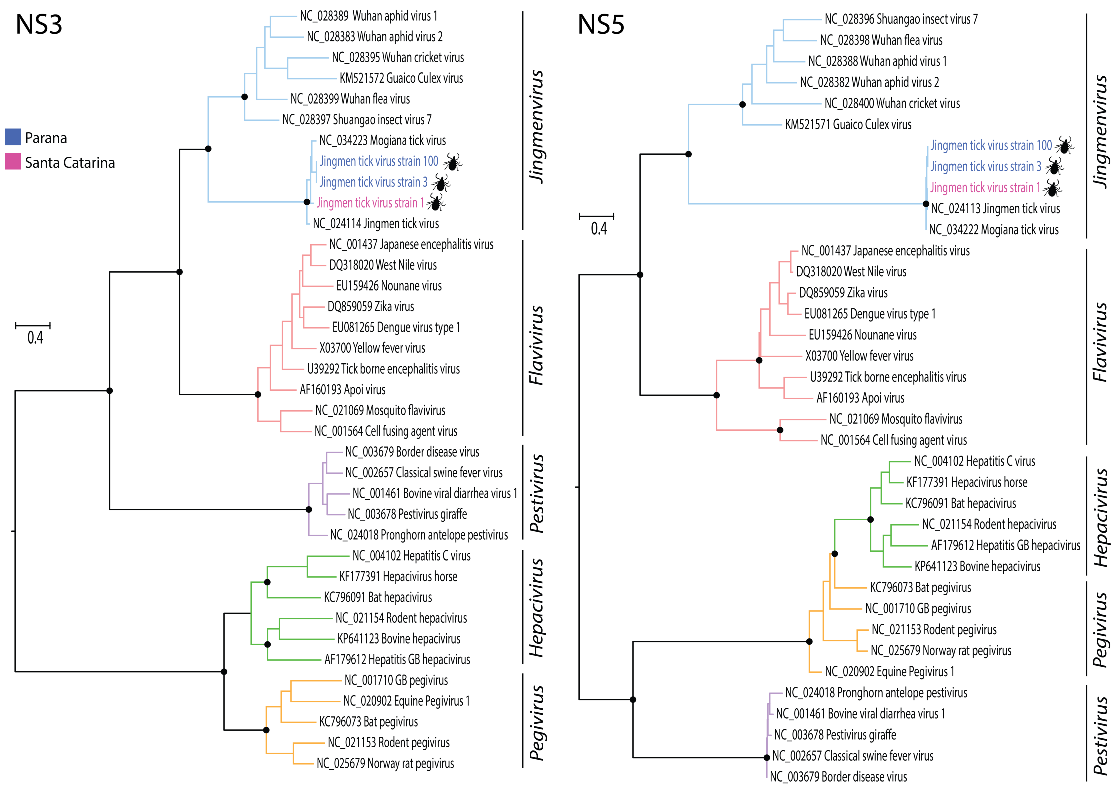

Supplementary Figure 2. Genome organization of Jingmen tick virus (a), Lihan tick virus (b) and Wuhan tick virus 2 (c).

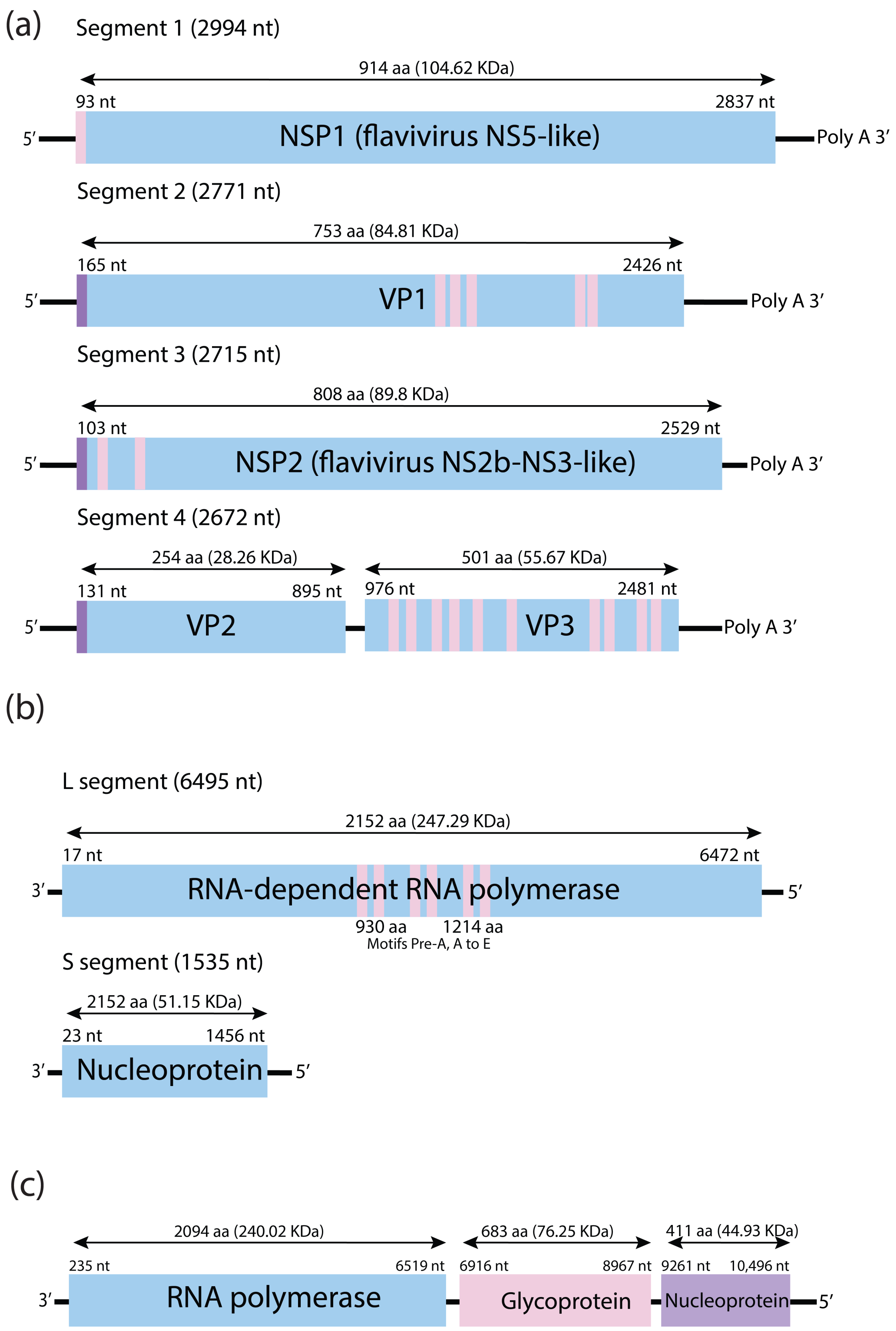

Supplementary Table

Supplementary Table 1. Samples information, host, location, year of collection, reads, and SRA accession number.

ID	Host	Location	Year	N	# Reads	SRA
WM100	*Rhipicephalus microplus*	Guarapuava, PR	2015	~50	26,880,904	SRR6848869
WM1	*Rhipicephalus microplus*	Lages, SC	2016	~50	18,439,774	SRR6848868
WM2	*Bos taurus*	Lages, SC	2016	4	18,294,452	SRR6848876
WM3	*Rhipicephalus microplus*	Manoel Ribas, PR	2016	~50	34,753,840	SRR6848867
WM4	*Bos taurus*	Manoel Ribas, PR	2016	15	7,634,294	SRR6848866
WM5	*Rhipicephalus microplus*	Guarapuava, PR	2016	~50	23,781,512	SRR6848873
WM6	*Bos taurus*	Guarapuava, PR	2016	5	18,122,532	SRR6848872
WM9	*Rhipicephalus microplus*	Ronda Alta, RS	2016	~50	24,293,740	SRR6848871
WM10	*Bos taurus*	Ronda Alta, RS	2016	6	21,850,234	SRR6848870
WM11	*Rhipicephalus microplus*	Ronda Alta, RS	2016	~50	18,100,514	SRR6848875
WM12	*Bos taurus*	Ronda Alta, RS	2016	6	16,910,852	SRR6848874

N: number of individual per pools.

Supplementary Table 2. Distribution of reads assembled by MetaViC per organism.

ID	Eukaryote	Bacteria	Viruses	Archaea	Unassigned
WM100	70% (2608)	6% (221)	24% (908)	-	0.2% (8)
WM1	40% (1846)	57% (129)	3% (133)	-	-
WM2	34% (431)	63% (811)	0.6% (8)	0.4% (5)	1% (14)
WM3	55% (22440)	40% (16134)	5% (2050)	-	-
WM4	30% (739)	69% (1710)	1%(24)	-	-
WM5	52% (16460)	45% (13938)	3% (1069)	-	-
WM6	41% (261)	54% (337)	0.8% (5)	0.6% (4)	3% (22)
WM9	22% (2440)	77% (8472)	1% (114)	-	-
WM10	49% (502)	46% (467)	0.6% (6)		4% (38)
WM11	34% (4265)	65% (8213)	1% (178)	-	-
WM12	19% (60)	78% (250)	1% (4)	-	-

Numbers of reads are showed between parentheses.

Supplementary Table 4. Primers used in RT-PCR described in this study.

Virus		Primer name	Sequence (5'to3')	Amplicons	Reference
Jingmen tick virus	Outer primer	Segment1_1437F	TCGGCGATAAATAGGAGAGGTGCCAT	486 pb	Qin et al., 2014
		Segment1_1923R	TCTGCGTAGAGTCGGTAGAGGTGGTG		
	Inner primer	Segment1_1520F	GGACTGGAGACAAGACGTCAACACG	370 pb	
		Segment1_1890R	CGCCATTTCTTCATCCTCCGCTAG		
Lihan tick virus	-	LTV-Forward	GCATCTCGGATCAATAACCT	894 pb	Our study
		LTV-Reverse	CATACACCCCCTCTTCTCTA		
Wuhan tick virus 2	-	WTV2-Forward	CATTAGCACAGCAGTTTCAG	991 pb	Our study
		WTV2-Reverse	GCATGTTTGAGCATACGAAA		
